# Delayed apoptosis by neutrophils from COPD patients is associated with altered bak, bcl-xl, and mcl-1 mRNA expression

**DOI:** 10.1186/1746-1596-7-65

**Published:** 2012-06-11

**Authors:** Jisong Zhang, Jian He, Jingwen Xia, Zhen Chen, Xiaodong Chen

**Affiliations:** 1 Department of Pulmonology, Huashan Hospital, Fudan University, Shanghai, 200040, China; 2 Hangzhou Xiasha Hospital, Hangzhou, China

**Keywords:** Apoptosis, Bcl-2, Chronic obstructive pulmonary disease, Lung function, Neutrophils

## Abstract

**Background:**

Delayed neutrophil apoptosis may be an important factor in the persistent inflammation associated with chronic obstructive pulmonary disease (COPD). Bcl-2 family proteins are important regulators of neutrophil apoptosis. We determined the mRNA levels of pro-apoptotic Bak and anti-aptototic Bcl-xl and Mcl-1 members of the Bcl-2 family in unstimulated peripheral blood neutrophils from patients with mild to moderate COPD and compared these to neutrophils from healthy controls.

**Methods:**

Neutrophils were isolated from peripheral blood samples of 47 COPD patients (smokers: N = 24) and 47 healthy controls (smokers: N = 24). Percentages of apoptotic cells were determined at 4, 24, and 36 h for unstimulated neutrophils cultured *in vitro*. Neutrophil mRNA expression of Bak, Bcl-xl, and Mcl-1 was determined by real-time polymerase chain reaction (PCR). FEV1 (% predicted) and FVC were determined by spirometry and correlations between mRNA levels and lung function parameters were determined.

**Results:**

The percentages of apoptotic cells among unstimulated neutrophils from COPD patients were significantly lower compared to cells from controls after 4, 24, and 36 h in culture; smoking history had only a minimal effect on these differences. Unstimulated neutrophils from COPD patients had significantly lower Bak mRNA expression and higher expressions of Bcl-xl and Mcl-1 mRNA than cells from healthy controls. Again, smoking history had only a minimal effect on these trends. Bak mRNA expression was significantly positively correlated with both % predicted FEV1 and the FEV1/FVC ratio, while Bcl-xl and Mcl-1 mRNA expressions were significantly negatively correlated with %predicted FEV1 and the FEV1/FVC ratio.

**Conclusions:**

The genes for pro-apoptotic Bak, and anti-apoptotic Bcl-xl and Mcl-1 may be important in regulating the delayed neutrophil apoptosis observed in COPD, which may contribute to COPD pathogenesis.

**Virtual Slides:**

The virtual slide(s) for this article can be found here: http://www.diagnosticpathology.diagnomx.eu/vs/1605269445677066

## Introduction

Chronic obstructive pulmonary disease (COPD) is characterized by irreversible airflow limitation [1]. COPD is also characterized by an abnormal inflammatory response to noxious gases, especially cigarette smoke, and these abnormal inflammatory responses exacerbate airflow obstruction [2]. An inflammatory pulmonary infiltrate comprised primarily of neutrophils is important in COPD pathogenesis. Delayed cellular apoptosis can prolong the life of neutrophils and can lead to their accumulation, resulting in a persistent inflammation in the lungs and airway, which is considered to be a critical step in COPD pathogenesis [3]. Aggregation of neutrophils in the lungs and airway is another characteristic of COPD [4-6].

Although a number of studies have confirmed the delayed apoptosis of neutrophils in the peripheral circulation, and in induced sputum and bronchoalveolar lavage fluid (BALF) samples during the development of COPD [7-10], the mechanisms underlying this phenomenon are not clear. One report found no relationship between serum or sputum cytokines and the rate of neutrophil apoptosis during COPD exacerbations [11]. However, Brown et al. found that neutrophil apoptosis was reduced in both stable COPD subjects and in healthy controls who were smokers, which might have been attributed to the activation of NFκB [12].

Some genes may increase the risk of COPD development in certain populations. Ning et al. found 6 genes whose expressions were different in mild COPD patients who were smokers and in smokers without lung injury [13]. Thus, we hypothesized that delayed neutrophil apoptosis was caused by differences in the expressions of genes involved in the apoptotic regulatory pathway.

Members of the Bcl-2 protein family are part of the mitochondrial intrinsic apoptotic pathway, and it has been suggested that regulation of neutrophil apoptosis primarily involves members of this family [14]. Human neutrophils express many members of the Bcl-2 family, including the pro-apoptotic proteins Bax, Bid, Bak, and Bad, and the anti-apoptotic proteins Mcl-1, A1, and, Bcl-xl [15,16].

We hypothesized that a reason for the delayed neutrophil apoptosis observed in COPD is the abnormal expression of Bcl-2 family members. For this study, we selected genes for the pro-apoptotic protein Bak and the anti-apoptotic proteins Bcl-xl and Mcl-1, as they have been considered to be critical genes in regulating neutrophil apoptotic pathways [14,15]. We examined the mRNA expressions of these genes in unstimulated peripheral blood neutrophils from patients with mild to moderate COPD and healthy volunteers to investigate the relationship between Bcl-2 family gene regulation, delayed neutrophil apoptosis, and COPD pathogenesis.

## Methods

### Subjects

All COPD patients in this study were in a stable stage and were recruited from the out-patient clinic of our hospital. These patients had mild-to-moderate COPD (total: n = 47; smokers: n = 24). The categorization of mild-to-moderate COPD was based on the Global Initiative on Chronic Obstructive Lung Disease (GOLD) criteria (http://www.goldcopd.org). Patients who were possibly asthmatic were excluded based on the criteria of the Global Initiative on Asthma (GINA; http://www.ginaasthma.org).

We also recruited age-matched healthy controls (total: n = 47; smokers: n = 24). For both COPD patients and healthy controls, smoking history was recorded in pack-years. Exclusion criteria for all subjects were history of allergy, tuberculosis, neoplasm, asthma, thoracic or abdominal surgery, or other serious concomitant diseases. No subjects had had an infection of the respiratory tract for at least 3 months, and none were currently being treated with antibiotics or steroids. The study was approved by the ethics review committee of Huashan Hospital, Fudan University and was conducted in accordance with the Declaration of Helsinki and Good Clinical Practice guidelines. All patients gave written informed consent.

### Lung function determinations

Lung function was determined in the Lung Function Laboratory according to standard protocols using a pneumotachograph (Jaeger MS Diffusion, Würzburg, Germany). A subject breathed smoothly several times, and then inspired forcibly until total lung capacity was achieved. Subsequently, the subject expired forcibly until reaching his/her residual capacity. Then, the subject inspired until total lung capacity was achieved followed by expiration. Results for the % predicted forced expiratory volume in one second (FEV1) and the forced vital capacity (FVC) were generated automatically.

COPD patients were asked to stop taking long-acting aminophylline 48 h or oral aminophylline 12 h before the examination and stopped using any inhaled β−agonist 6 h before the examination. If the FEV1/FVC was < 70%, patients were recommended to inhale salbutamol (Ventolin) (200 μg × 2 puffs), and spirometry was repeated 15 min later. An increase in FEV1/FVC of ≥ 12% and an absolute increase of FEV1 ≥ 200 ml were regarded as positive for bronchodilation.

### Isolation of peripheral blood neutrophils

Neutrophils were isolated from peripheral blood samples collected in sterile heparinised tubes by a two-step procedure. First, erythrocytes were removed by sedimentation using dextran (T500, Pharmacia). Next, granulocytes were isolated using discontinuous Percoll gradient centrifugation [17]. We used 0.9% saline to prepare 60% and 70% (by volume) Percoll solutions (densities of 1.079 g/ml and 1.091 g/ml, respectively) from a stock solution of 1.130 g/ml (100% fine grade Percoll, Pharmacia). After centrifugation, the density of peripheral blood granulocytes, primarily neutrophils, in the Percoll solution was between 1.080 to 1.085 g/ml. The isolated cells were determined to be > 98% neutrophils by Giemsa staining.

### Neutrophil apoptosis assay

Neutrophils were suspended to a concentration of 5 × 10^5^cells/ml in RPMI 1640 medium supplemented with 10% FCS and 100 U/ml of penicillin-streptomycin, and 200 μl of this suspension was pipetted into each well of a 36 well flat-bottom microtest plate. Unstimulated cells were cultured at 37°C in a 5% CO_2_ atm for 4, 24, or 36 h, after which the percent of apoptotic cells was determined using previously described methods [18]. Briefly, after the indicated culture time, cells were stained with a FITC-Annexin V antibody (Abcam) followed by staining with propidium iodide (PI). After washing in PBS, cells were evaluated by flow cytometry using a FACScan (Becton-Dickinson, San Jose, CA). Forward and side scatter gates were set to evaluate neutrophils (granulocytes) only. The percent of cells that were double-positive for Annexin V and PI were recorded as percent apoptotic cells.

### RNA extraction from neutrophils and reverse transcription (RT)-PCR

Total RNA was extracted from neutrophils with RNAisoTMPlus (TaKaRa Biotechnology) and reverse transcribed using PrimeScriptTM RT reagent kits (TaKaRa Biotechnology). Reactions mixtures were 8 μL of total RNA, 4 μL of 5 × PrimeScriptTM Buffer, 1 μL of PrimeScriptTMRT Enzyme Mix I, 1 μL of oligo dT primer, 1 μL of random primer, and 5 μL of RNase-free deionised H_2_O.

### Real-time quantitative PCR

Neutrophils’ expressions of Bak, Bcl-xl, and Mcl-1 mRNA were determined by real-time quantitative PCR [19]. Real-time PCR used an Applied Biosystems 7500 Fast Real-Time PCR System with glyceraldehyde-3-phosphate dehydrogenase (GAPDH) as the internal control. The gene sequences for Bak, Bcl-xl, Mcl-1, and GAPDH were from the NCBI database (http://www.ncbi.nlm.nih.gov/). Primer Express Version 3.0 (Applied Biosystems, America) was used to design specific primers (details in Table 1). SYBR Green Master Mix (Applied Biosystems, America) was used for real-time PCR, with initial denaturation at 95°C for 10 min, followed by 40 cycles at 95°C for 15 s, 60°C for 30 s, and 72°C for 30 s. An identical threshold cycle (Ct) was used for each gene of interest. Relative mRNA expression levels were determined according to the 2^-ΔΔCT^ method [20].

**Table 1:** Nucleotide primer sequences used for real-time quantitative PCR

**Target genes**	**Nucleotide sequence**	**Orientation**	**Amplicon length**	**Accession No.**
Bak	5^′^— CCCAGGACACAGAGGAGGTTT—3^′^ 5^′^— GCCTCCTGTTCCTGCTGATG—3^′^	Sense Antisense	65 bp	NM001188
Bcl-xl	5^′^— TGCGTGGAAAGCGTAGACAA—3^′^ 5^′^—ATTCAGGTAAGTGGCCATCCAA—3^′^	Sense Antisense	75 bp	NM138578
Mcl-1	5^′^— AGGCTGGGATGGGTTTGTG—3^′^ 5^′^—CACATTCCTGATGCCACCTTCT—3^′^	Sense Antisense	65 bp	NM021960
GAPDH	5^′^- AACAGCCTCAAGATCATCAGCA-3^′^ 5^′^- CATGAGTCCTTCCACGATACCA-3^′^	Sense Antisense	102 bp	M33197

### Statistical analysis

Results for subjects’ demographic and clinical characteristics are given as means ± standard deviations (SD) for continuous variables and n (%) for categorical variables. Results for mRNA expression and apoptosis percentages are given as means ± standard errors of the mean (SEM). For continuous variables, groups were compared using a two-sample *t*-test or a Mann–Whitney *U* test if data were not normally distributed. A Pearson chi-square test was used for categorical variables. Spearman correlation analysis was used to evaluate associations between mRNA expression levels and lung function parameters. Statistical significance was set at P < 0.05. Statistical analyses used SPSS 15.0 software (SPSS, Inc, Chicago, IL, USA).

## Results

### Study subject characteristics

A total of 94 subjects were enrolled in this study. The demographic and clinical characteristics of the healthy controls (n = 47) and COPD patients (n = 47) are shown in Table 2. There were more males than females (40 vs. 7 in each group), and the COPD patients were significantly older, on average, than the controls (61.3 ± 4.5 years vs. 58.8 ±4.6 years; P = 0.011). Slightly more than 50% of the subjects were smokers in both groups. Thirty (63.8%) COPD patients used inhaled short-acting bronchodilators as needed and, among these patients, 18 also used inhaled long-acting bronchodilators.

**Table 2:** Subjects’ demographic and clinical characteristics

**Variables**	**Healthy controls (n = 47)**	**COPD patients (n = 47)**	**P-value**
Age (years) ^†^	58.8 ± 4.6	61.3 ± 4.5	0.011^*^
Sex^‡^			1.000
Males	40 (85.1%)	40 (85.1%)	
Females	7 (14.9%)	7 (14.9%)	
Smokers^‡^	24 (51.1%)	24 (51.1%)	1.000
Smoking history (pack/year) ^§^	31.9 ± 7.4	31.7 ± 6.0	0.755
FEV_1_/FVC %^†, §^	86.6 ± 4.5	56.8 ± 5.3	<.001^*^
FEV_1_,% Reference^†, §^	103.1 ± 9.5	59.4 ± 6.3	<.001^*^
ISAB as needed^‡^	0 (0)	30 (63.8)	-
ILAB regularly^‡^	0 (0)	18 (38.3)	-

Abbreviations: COPD: chronic obstructive pulmonary disease; ISAB: inhaled short-acting bronchodilators; ILAB: inhaled long-acting bronchodilators.

^†,‡,§^ Results are means ± SDs for ^†,§^continuous variables and n (%) for ^‡^categorical variables.

^†‡§^ Groups were compared using ^†^two-sample *t*-test or ^§^Mann–Whitney *U* test if data were not normally distributed. ^‡^Pearson Chi-square test was used for categorical variables.

^*^ P < 0.05, significantly different between groups.

### Neutrophil apoptosis

**Figure 1:**
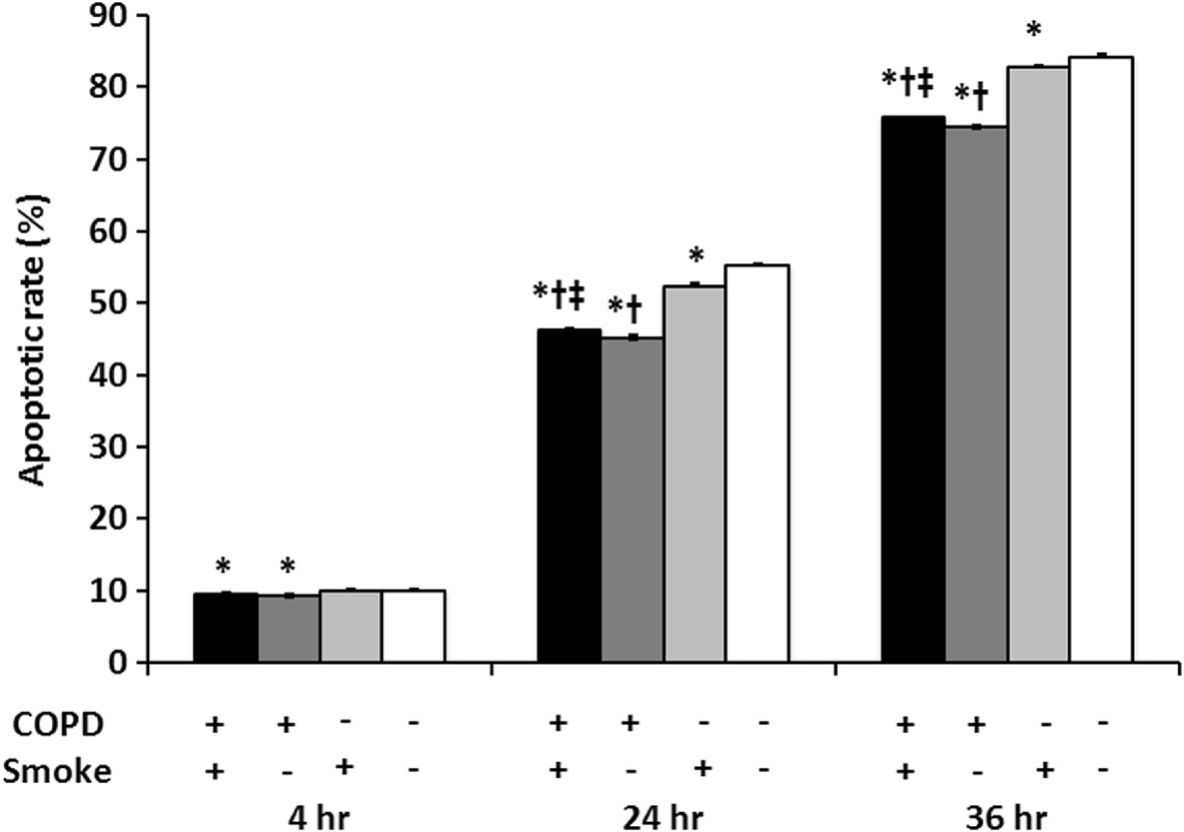
**Comparisons of percentages of apoptotic neutrophils from healthy controls and COPD patients.** Peripheral blood neutrophils were isolated from healthy controls and COPD patients; these groups were sub-divided into those with and without smoking histories. As described in Methods, unstimulated peripheral blood neutrophils were cultured *in vitro* for 4, 24, or 36 h, after which the percent of apoptotic cells was determined. Results are means ± SEMs for: COPD/smokers (n = 24); COPD/non-smokers (n = 23); Healthy/smokers (n = 24); and Healthy/non-smokers (n = 23). Group comparisons were by Mann–Whitney *U* test. * significant difference as compared with Healthy/non-smokers (p < 0.05); † significant difference as compared with Healthy/smokers (p < 0.05); ‡ significant difference as compared with COPD/non-smokers (p < 0.05).

Apoptosis was determined for unstimulated neutrophils in culture. Figure 1 shows the percentages of apoptotic peripheral blood neutrophils from COPD patients and healthy controls after different times in culture (4, 24, and 36 h.). Each subject group was also sub-divided into those with smoking and non-smoking histories. Compared to neutrophils from healthy controls, the percentages of COPD patients’ apoptotic neutrophils were significantly lower after culture for 4, 24, and 36 h. A smoking history slightly increased the percent of COPD patients’ apoptotic neutrophils, although the percent of apoptotic cells was still lower than for cells from controls who smoked. These results show that unstimulated neutrophils from COPD patients have significantly delayed apoptosis relative to neutrophils from healthy controls.

### Neutrophil mRNA expression of apoptosis-related genes

**Figure 2:**
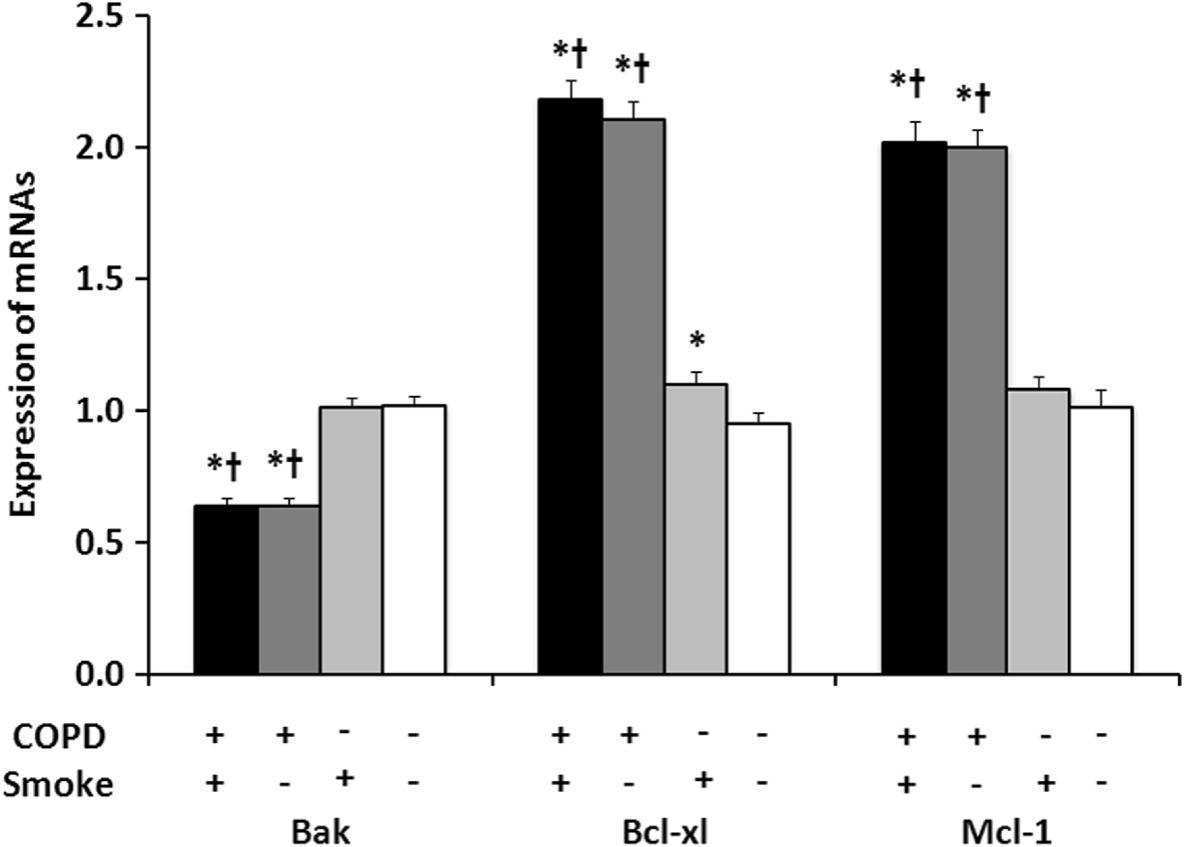
**Expression of Bak, Bcl-xl, and Mcl-1 mRNA’s in peripheral blood neutrophils.** Real-time PCR was used to determine the expression of (**A**) Bak, (**B**) Bcl-xl, and (**C**) Mcl-1 mRNAs in peripheral blood neutrophils. The ΔΔCt value for each mRNA was normalized to the value of the GADPH housekeeping gene mRNA. Results are means ± SEMs for: COPD/smokers (n = 24); COPD/non-smokers (n = 23); Healthy/smokers (n = 24); and Healthy/non-smokers (n = 23). Groups were compared using two-sample *t*-test for Bak and Mcl-1 mRNA’s, and a Mann–Whitney *U* test for Bcl-x1 mRNA due to its non-normal distribution. * significant difference as compared with Healthy/non-smokers (p < 0.05); † significant difference as compared with Healthy/smokers (p < 0.05).

Figure 2 shows the relative expressions of Bak, Bcl-xl, and Mcl-1 mRNA’s by unstimulated peripheral blood neutrophils that were analyzed using real-time PCR. In neutrophils from COPD patients, Bak mRNA expression was significantly reduced and the expressions of Bcl-xl and Mcl-1 mRNA’s were significantly increased compared to neutrophils from controls. As with neutrophil apoptosis, a history of smoking had an effect on mRNA expression; however, these differences between mRNA expressions remained significantly different between COPD patients and controls regardless of smoking history. In COPD patients’ neutrophils, mRNA expression for the two anti-apoptotic genes, Bcl-xl and Mcl-1, was almost twice that of controls (Bcl-xl: 2.1 ± 0.3 vs. 1.0 ± 0.2; Mcl-1: 2.0 ± 0.4 vs. 1.0 ± 0.3; both P < 0.001).

### Lung function and neutrophil mRNA expression of apoptosis-related genes

**Figure 3:**
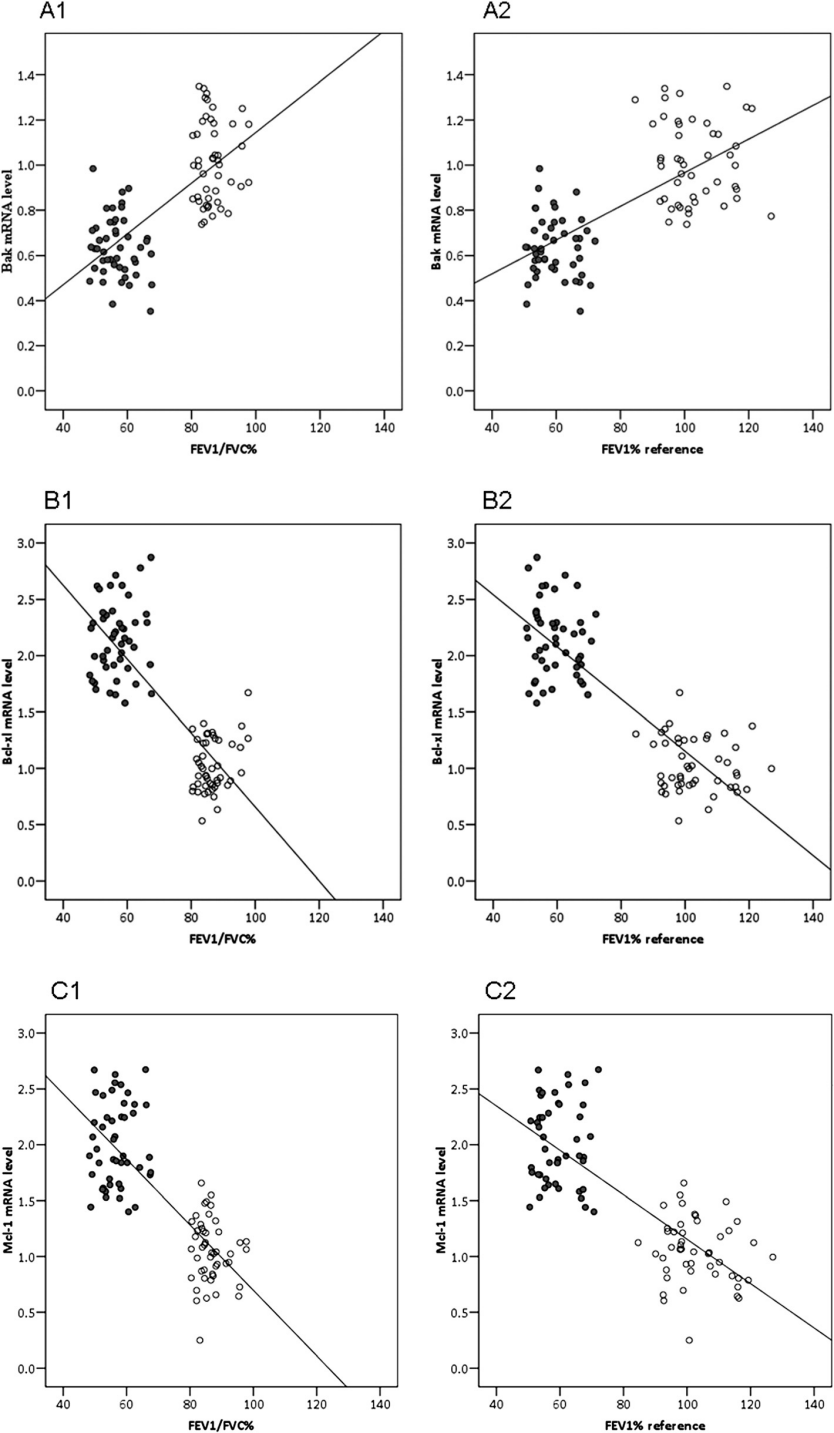
**Correlations between mRNA levels of Bak, Bcl-xl, and Mcl-1 and lung function parameters.** COPD patients are represented by solid circles and healthy controls by open circles (N = 47 for each group) Spearman correlation analysis was used to assess associations between variables. (**A**) Bak mRNA level was positively correlated with lung function. FEV1/FVC%: r = 0.72; P < 0.001; FEV1% reference: r = 0.70; P < 0.001. (**B**) Bcl-xl mRNA level was negatively correlated with lung function. FEV1/FVC%: r = -0.82; P < 0.001; FEV1% reference: r = -0.86; P < 0.001. (**C**) Mcl-1 mRNA level was negatively correlated with lung function. FEV1/FVC%: r = -0.79; P < 0.001; FEV1% reference: r = -0.79; P < 0.001.

As shown in Table 2, spirometric lung function parameters were significantly worse for COPD patients than for healthy controls, including % predicted FEV_1_ and the FEV_1_/FVC ratio. We used Spearman correlation analysis to determine if these lung function parameters were associated with neutrophils’ mRNA expression of apoptosis-related genes. Figure 3 shows the correlations between the mRNA expression levels for Bak (Figure 3A), Bcl-xl (Figure 3B), and Mcl-1 (Figure 3C) and lung function. These correlation results were not affected by smoking status (data not shown); thus, these correlations are shown by COPD status only without stratifying according to smoking status. Bak mRNA levels were significantly positively correlated with lung function parameters, and Bcl-xl and Mcl-2 mRNA levels were significantly negatively correlated with lung function parameters.

## Discussion

The aim of this study was to investigate whether changes in gene expressions for members of the Bcl-2 family of pro- and anti-apoptotic proteins were involved in the delayed neutrophil apoptosis seen in COPD. We found that, in peripheral blood neutrophils from subjects with stable mild to moderate COPD, mRNA expressions for two anti-apoptotic genes (Bcl-xl and Mcl-1) were upregulated and that for a pro-apoptotic gene (Bak) was downregulated (Figure 2). Our findings showed that the abnormal expression of Bcl-2 family member genes was present in an early stage COPD and that this trend of abnormal gene expression was consistent with the delayed apoptosis seen in neutrophils from COPD patients (Figure 1). We also found that these mRNA expression levels were significantly correlated with lung function parameters (Figure 3).

In our study, after 36 h in unstimulated culture, the apoptosis rate of peripheral blood neutrophils of COPD patients was significantly delayed relative to neutrophils from healthy controls. For peripheral blood neutrophils, others have reported decreased apoptosis during COPD exacerbations, but not in stable COPD [8,9]. Using cells from sputum samples, neutrophil apoptosis for COPD patients who were current smokers was reported to be only one-third of that seen in sputum samples from healthy non-smokers.^12^ This difference in results between neutrophils from peripheral blood and sputum is presumably due to the additional effect on sputum neutrophils of the chronic inflammation present in the lungs of COPD patients, particularly those who smoke.

In our study, the differences between COPD patients and controls in neutrophil mRNA levels of Bcl-2 family genes were greater than the differences seen in the percentages of apoptotic neutrophils. The mRNA levels for the two anti-apoptotic genes (Bcl-xl and Mcl-1) were twice as high as in controls, and the mRNA level of the pro-apoptotic gene (Bak) was 60% of that in controls’ neutrophils.

The Bcl family contains pro- and anti-apoptotic proteins that are found in the outer membranes of mitochondria. The balance between the pro- and anti-apoptotic members of this family is the central factor in the opening and closing of permeability transition (PT) pores [21], which is a critical pathway for cytochrome c release from mitochondria [22] and causes apoptosis by activating caspase-9. The increase in anti-apoptotic mRNA expression and decrease in pro-apoptotic mRNA expression seen in our study could tip the balance toward neutrophil survival and decrease the apoptosis rate.

However, it is the relative abundance of Bcl-2 family proteins, rather than their mRNA expression, that would ultimately regulate apoptosis, and protein levels do not always correspond to mRNA levels. This was reportedly the case for Bcl-xL. Moulding et al. [15], who studied peripheral blood neutrophils from normal subjects, detected Bcl-xL mRNA in these cells, but could not detect the corresponding protein. They also found that blocking gene transcription with actinomycin decreased Mcl-1 mRNA and protein, but had virtually no effect on Bcl-xL mRNA levels. Therefore, Bcl-xL may not be involved in the control of apoptosis in human neutrophils.

Although gene transcription is important in determining mRNA levels, the mRNA half-life is also important. The half-life of the mRNA for Mcl-1, an anti-apoptotic protein, is relatively short and the half-life of the mRNA for Bak, a pro-apoptotic protein, is relatively long [15]. Thus, Mcl-1, whose mRNA levels can change relatively rapidly, might be more important than Bak in the short-term control of apoptosis. A relationship between decreased Mcl-1 mRNA expression and an increase in neutrophil apoptosis has previously been reported for patients with sepsis [23], but until now, research on COPD patients has been lacking.

We found only minimal differences in Bcl-2 family mRNA expression between healthy smokers and healthy non-smokers, and between smoking and non-smoking COPD patients. This suggests that the effects of smoking are not evident in neutrophils from peripheral blood as would be expected in cells from BALF, especially in patients with mild to moderate COPD like those included in the current study. An alternative explanation would be that Bcl-2 member expression is implicated in COPD pathogenesis, but not through a pathway that is affected by smoking. In addition, COPD is the result of the interactions between genes and the environment [24,25]. Genes other than Bcl-2 family members could be affected by these factors.

More studies are needed to investigate the relationships between mRNA and protein levels, and to determine whether the results of Bcl family members in normal neutrophils apply to COPD patients. An in-depth study is also needed on the neutrophil apoptosis pathways that are influenced by cigarette smoking and the complex effects of nicotine on neutrophil apoptosis *in vivo*.

## Conclusions

We evaluated associations between Bcl-2 family members mRNA expressions and delayed apoptosis for unstimulated peripheral blood neutrophils from COPD patients. Relative to neutrophils from healthy controls, pro-apoptotic gene Bak mRNA was significantly downregulated in COPD patients’ neutrophils, whereas anti-apoptotic gene Bcl-xl and Mcl-1 mRNA’s were significantly upregulated. In addition, Bak mRNA was significantly positively correlated with %predicted FEV1 and the FEV1/FVC ratio, while Bcl-xl and Mcl-1 mRNA’s were negatively correlated with these parameters. The trend for abnormal mRNA expression for members of the Bcl-2 family was consistent with the decline in lung function in COPD. Therefore, the most likely neutrophil apoptosis pathway in COPD patients may be the mitochondrial pathway, which could be a new target for future COPD research.

## Competing interests

The author(s) declare that they have no competing interests.

## Authors' contributions

JZ, JH: experimental studies, writing the paper; JX: manuscript editing; ZC: experimental guidance; XC: guarantor of integrity of the entire study. All authors read and approved the final manuscript.
